# Behavioral and Physiological Changes during Benthic-Pelagic Transition in the Harmful Alga, *Heterosigma akashiwo*: Potential for Rapid Bloom Formation

**DOI:** 10.1371/journal.pone.0076663

**Published:** 2013-10-04

**Authors:** Elizabeth D. Tobin, Daniel Grünbaum, Johnathan Patterson, Rose Ann Cattolico

**Affiliations:** 1 School of Oceanography, University of Washington, Seattle, Washington, United States of America; 2 Department of Biology, University of Washington, Seattle, Washington, United States of America; Mount Allison University, Canada

## Abstract

Many species of harmful algae transition between a motile, vegetative stage in the water column and a non-motile, resting stage in the sediments. Physiological and behavioral traits expressed during benthic-pelagic transition potentially regulate the timing, location and persistence of blooms. The roles of key physiological and behavioral traits involved in resting cell emergence and bloom formation were examined in two geographically distinct strains of the harmful alga, *Heterosigma akashiwo*. Physiological measures of cell viability, division and population growth, and cell fatty acid content were made using flow cytometry and gas chromatography – mass spectrometry techniques as cells transitioned between the benthic resting stage and the vegetative pelagic stage. Video-based tracking was used to quantify cell-level swimming behaviors. Data show increased temperature and light triggered rapid emergence from the resting stage and initiated cell swimming. Algal strains varied in important physiological and behavioral traits, including survivorship during life-stage transitions, population growth rates and swimming velocities. Collectively, these traits function as “population growth strategies” that can influence bloom formation. Many resting cells regained the up-swimming capacity necessary to cross an environmentally relevant halocline and the ability to aggregate in near-surface waters within hours after vegetative growth supporting conditions were restored. Using a heuristic model, we illustrate how strain-specific population growth strategies can govern the timescales over which *H. akashiwo* blooms form. Our findings highlight the need for identification and quantification of strain-specific physiological and behavioral traits to improve mechanistic understanding of bloom formation and successful bloom prediction.

## Introduction

Harmful algal blooms (HABs) occur when algal cells produce toxins or accumulate to densities sufficient to be deleterious to other organisms, causing damage to aquatic ecosystems and/or risks to public health [1-4]. Many behavioral and physiological functional traits that regulate HAB dynamics remain poorly understood. Improved understanding of these traits to help manage and mitigate HAB impacts is a current priority of basic and applied research [5].

Many HAB-forming species exhibit a dual-stage life history, in which they alternate between a pelagic vegetative stage and a benthic resting stage (e.g., cysts, resting spores or temporary resting cells). Transitions between these stages have potentially important impacts on bloom dynamics. Rapid transition of pelagic cells into the benthic resting stage can contribute to HAB termination [6,7]. Conversely, some HABs are thought to initiate when benthic cells return to the vegetative state and rapidly repopulate the water column [8,9]. This process typically requires benthic cells to increase metabolic activity, to emerge from the sediments and ascend toward the surface of the water column, and finally to undergo rapid cell division to form population densities characteristic of blooms. Despite this potentially causal role in bloom dynamics, life stage transitions are among the least understood aspects of HAB dynamics.

Cell transitions between benthic and pelagic environments often include depth changes that are associated with changes in environmental characteristics (e.g., depth, temperature and light) and may significantly influence diverse aspects of algal cell biology. One aspect involves cell swimming behaviors. Many HAB-forming algal species are capable of rapid vertical migration (e.g., tens of meters within 24 hours) [10-12]. Because resting cells occupy benthic habitats that may not provide optimal conditions for cell division, vigorous swimming behaviors expressed during benthic-pelagic transition may be critical to cell survival by regulating vertical fluxes to the photic zone.

Cell physiology (e.g., metabolic processes and maintenance of energy reserves) represents another aspect of algal cell biology influenced by benthic-pelagic life stage transitions. Presently, little is known concerning the relationship between changing physiological cues and the metabolic requirements for either cell survival during the benthic resting stage, or for active swimming during benthic emergence. It is well established that polyunsaturated fatty acids (PUFAs) are essential in maintaining cellular membrane integrity and function during adverse changes in environmental conditions [13,14]. Neutral lipid reserves have been reported to provide an important energy source that supports algal motility [15]. These diverse contributions to cellular processes suggest the hypothesis that fatty acid content and composition play a central role in successful algal life stage transitions.

In this study, we examined physiological and behavioral traits thought to regulate benthic emergence and surface bloom formation in the harmful raphidophyte, *Heterosigma akashiwo* (Y. Hada). Blooms of this alga have been associated with fatalities of wild and pen-reared fish in temperate and sub-tropical waters [16,17]. Dense near-surface aggregations and rapid population growth are considered key determinants of the ecological impacts of *H. akashiwo* blooms [17-19]. *H. akashiwo* is capable of growing in salinities ranging from < 10 psu to 40 psu [20-22]. Cells exhibit vigorous up-swimming behavior in the vegetative stage [23] and readily swim across strong haloclines. In laboratory studies, Bearon et al. (2006) observed that *H. akashiwo* cells were capable of crossing a 28 to 8 psu halocline with only a modest decrease in swimming speeds. Halocline-crossing behavior has been hypothesized to be an important mechanism in bloom formation that promotes high-density surface aggregations [10,24,25]. Consistent with this hypothesis, *H. akashiwo* blooms often initiate in shallow coastal regions or inland marine waterways that are characterized by strong seasonal stratification [17,19,26-28].

In *H. akashiwo*, cell transition out of the benthic stage (“emergence”) is regulated largely by temperature and light [29]. Environmental observations suggest that the approximate lower limit for benthic emergence is 10°C, and that 15°C is required for rapid population growth [1,16,26,29-31]. Division rates of vegetative *H. akashiwo* cells are regulated by environmental conditions such as light, temperature, salinity and nutrient concentrations, and typically range from 0.2-1.0 divisions per day [22,32-35]. However, higher division rates (up to ~4.0 div day^-1^) have been reported [16].

Interstrain variability in vegetative cells of *H. akashiwo* has been observed for a suite of physiological and behavioral parameters (e.g., photosynthetic rates, salinity and temperature tolerance, nitrogen sourcing, growth rates, toxin production and swimming speeds [23,34,35]), suggesting that traits expressed during pelagic and benthic transitions may also be strain-specific. Selection among traits is often associated with metabolic trade-offs that may lead to diversity in survival strategies [36]. For this reason, we evaluated physiological and behavioral traits during benthic-pelagic transitions in two *H. akashiwo* strains that are distinct geographically (west Atlantic and east Pacific, respectively) and genetically (Black and Cattolico, unpublished data).

The specific goals of this study were: a) to quantify strain-specific rates of benthic emergence, population growth and up-swimming in *H. akashiwo*; b) to gain insight into the roles of fatty acid reserves in *H. akashiwo* benthic-pelagic transitions; and c) to integrate these traits into a conceptual framework to assess their implications for timescales of *H. akashiwo* bloom formation. To accomplish these goals, cell viability, cell division and population growth, fatty acid content and cell-level swimming were characterized and quantified as key physiological and behavioral traits in *H. akashiwo* resting cell emergence and bloom formation.

## Materials and Methods

### Resting cell induction/activation

*Heterosigma akashiwo* cultures, strains CCMP 452 (Narragansett Bay, RI, USA, 1952) and UWC 13.03 (Salish Sea, U.S.A/C.A, 2003), were maintained in 2.8 liter Fernbach flasks containing 1.0 liter f/2 medium without silica (f/2-Si) [37]. Cultures were grown to stationary phase (>2x10^5^ cells mL^-1^) at 20°C, on a 12 hour light, 12 hour dark (50 µmol m^-2^ s^-1^) photoperiod with continuous rotary agitation (60 rpm). These cells were used to inoculate 250 mL experimental flasks at a density of 1x10^4^ cells mL^-1^. Each flask contained 125 mL f/2-Si medium and were fitted with silicone sponge plugs (Bellco Glass, Vineland, NJ, USA). Triplicate sub-cultures for each strain were maintained at 15°C on a 16 hour light: 8 hour dark (50 µmol m^-2^ s^-1^) photoperiod. After 5 days at 15°C, cell concentrations within each experimental flask were approximately 2-4x10^4^ cells mL^-1^.

To induce *H. akashiwo* cells to enter the benthic resting stage, the experimental flasks were wrapped in aluminum foil and placed in the dark at 10°C for 18 days [29,38]. Resting cells were then signaled to “activate” (i.e., exit stasis) by unwrapping the experimental flasks and exposing them to a 16 hour light, 8 hour dark (20 µmol m^-2^ s^-1^) photoperiod and temperature of 12°C. These environmentally relevant activation conditions represent typical spring/summer bottom water conditions of shallow embayments (< 20 m) within the Salish Sea (Department of Ecology, Marine Water Quality Monitoring Program: http://www.ecy.wa.gov/programs/eap/mar_wat/moorings.html). The experimental flasks were unwrapped during hour 6 of the light period (L6) and, therefore, were exposed to 10 hours of continuous light during the 1^st^ photoperiod.

### Experimental filming tank

**Figure 1:**
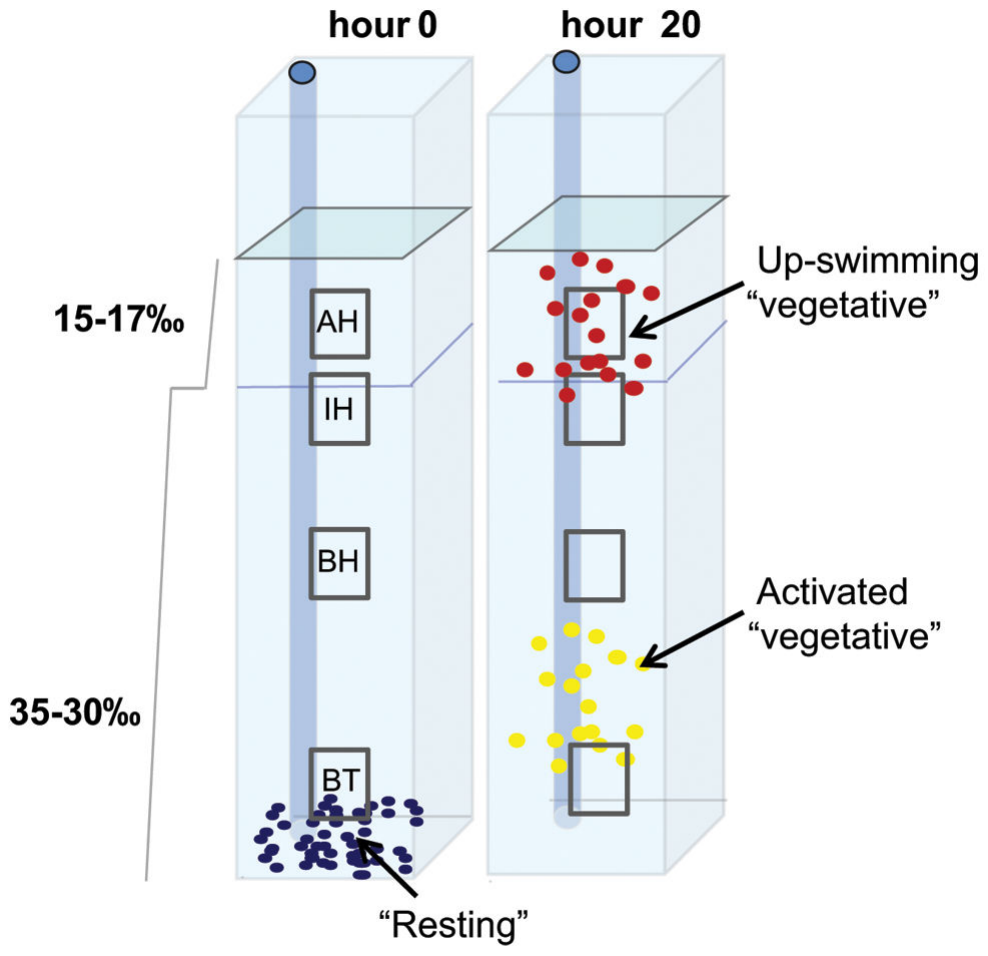
Schematics of the stratified water column within each replicate filming chamber of the experimental tank. Each chamber contained a two-layer water column separated by a 30-17 psu salinity transition (halocline). The four vertical fields of view are represented by the dashed boxes: bottom (BT), below the halocline (BH), in the halocline (IH) and above the halocline (AH). Schematics of initial (hour 0) and final (hour 20) cell distributions are shown.

Cells were placed in 6 equally spaced replicate filming chambers within a 300 mm x 240 mm x 50 mm experimental tank to observe cell swimming behaviors. Each chamber contained a two-layer stratified water column established using a peristaltic pump. A weak salinity gradient was established within each layer to suppress fluid motion. The top layer consisted of 100 mL f/2-Si medium diluted with double distilled fresh water to establish a salinity gradient of 17 to 15 psu, and the bottom layer had 350 mL diluted f/2-Si medium to form a gradient of 35 to 30 psu (Figure 1). The halocline formed between these two layers had a salinity jump of 13 psu.

On each observation day, 0.5-1 mL of the CCMP 452 resting cell culture and 2-4 mL of the UWC 13.03 resting cell culture were subsampled from each of the experimental flasks, and cells of each strain were added to 3 replicate filming chambers. Using a syringe, the cells were added slowly to the bottom of the tank through a fixed tube pre-positioned at the base of each chamber, ensuring that each chamber received approximately 1x10^4^ cells.

### Cell sampling for physiological measurements

**Figure 2:**
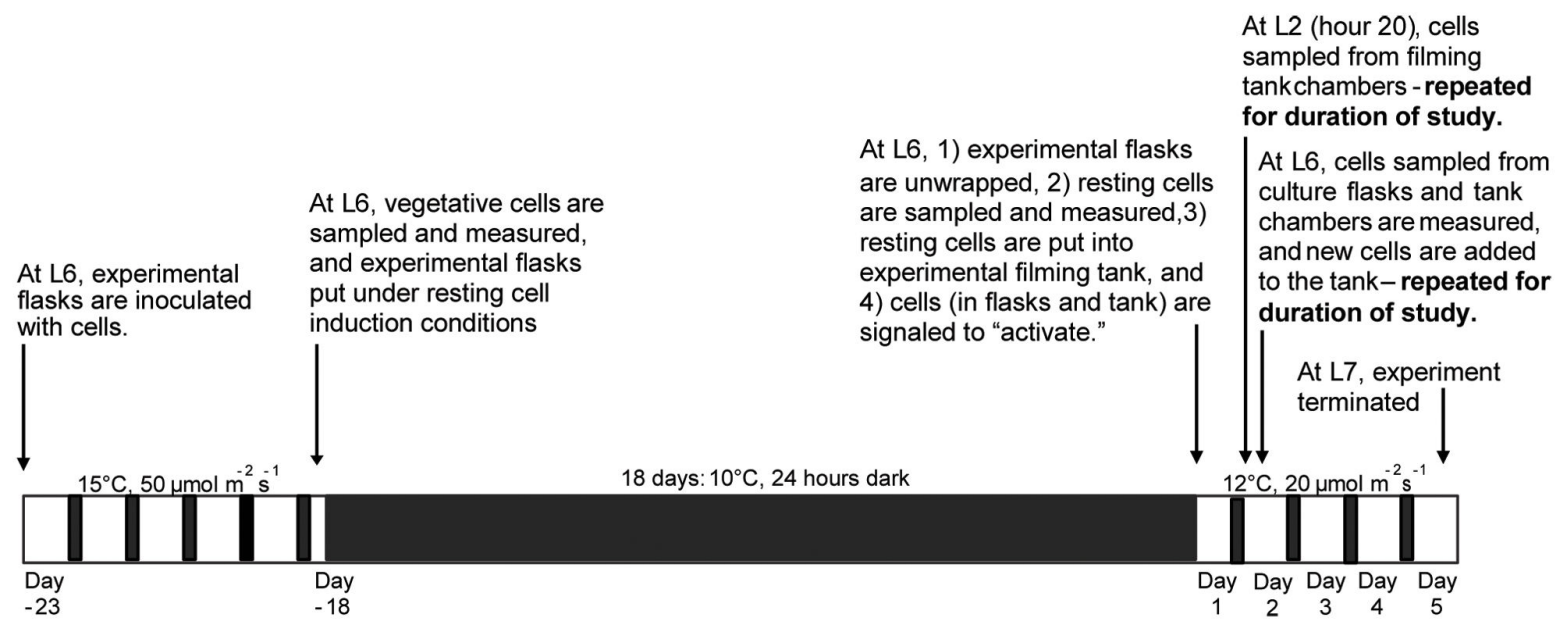
A timeline showing the environmental conditions and sampling periods over the duration of the study. The arrows indicate important sampling periods with an associated description. Experimental culture flasks were kept either in the dark for 24 hours per day, or on a 16 hour light: 8 hour dark photoperiod, as indicated on the timeline. “L6” refers to hour 6 of the light period.

Cells were subsampled from experimental culturing flasks to determine cell state (vegetative vs. resting), viability, population growth and neutral lipid content. Measurements on vegetative cells (“vegetative control”) were taken on cells subsampled just prior to induction into the resting stage (“day -18”). Resting cell measurements were taken after the 18 day induction period (“day 1”). Subsequent measurements were taken on subsamples of activated cell cultures (“days 2-5”) to monitor cell state, viability and population growth. Subsamples were collected consistently at L6 of the 16 hour light: 8 hour dark photoperiod (Figure 2). All measurements, except cell state, were made with a BD Accuri C6 flow cytometer (BD Biosciences, San Jose, CA, USA).

Cell state was determined under bright field microscopy (Zeiss Axioskop 2 plus, Carl Zeiss Microscopy, LLC, USA) under 400x magnification in a Palmer-Maloney counting chamber from subsamples recovered from the experimental flasks. Cells were classified as “vegetative” if they expressed propulsive helical swimming, irrespective of cell shape, or had no movement and were aspherical. Cells were classified as “resting” if they had no movement and were spherical [29,38].

### Cell viability and population growth

Cell viability was measured using the live/dead SYTOX-Green stain (Invitrogen Molecular Probes, Carlsbad, CA, USA). A 990 µL aliquot of subsampled cell culture into a 1.5 mL black microcentrifuge tube (Lite Safe Micro-Tubes, Research Products International Corp., Mount Prospect, IL, USA) containing 10 µL 50 µM SYTOX-Green working solution prepared by diluting 5 µL 5 mM SYTOX-Green solution with 495 µL f/2-Si medium. The microcentrifuge tubes were inverted 4 times and incubated for 10 min at 20°C. Flow cytometric measurements were made by adding 500 µL of the stained culture to a 5 mL glass test tube. Fluorescence of unstained cells was measured concurrently for background subtraction and to determine the cell concentration. Triplicate 500 µL samples were analyzed with the Accuri C6 flow cytometer. The SYTOX-stained samples were excited at 488 nm and emission was detected at 533 nm (±30 nm). Two wash cycles were run between sample types to eliminate cross-contamination.

Cell densities were measured from subsamples recovered from the experimental culture flasks and population density of viable cells was used to calculate specific growth rates (k) for each day after cells were signaled to activate (days 2-5; Table 1). Counts were also conducted on cell subsamples collected from each chamber of the experimental filming tank at the end of each 20-hour observation period (Figure 2). The entire 100 mL top layer was extracted from each chamber, gently mixed, and a 10 mL aliquot was collected. A 10 mL aliquot of the bottom layer was recovered from a fixed tube pre-positioned at the base of each chamber (Figure 1). The counts were used to determine the mean proportion of cells distributed within the bottom 10 mL and within the entire 100 mL top layer of the water column based on total number of viable cells added.

**Table 1:** Average measures of cell survival, life-stage transitions and population growth within the experimental flasks, and cell distributions within the experimental filming tank.

		**Flask**				**Tank**		
**Strain**	**Day**	**Resting (%)**	**Viable (%)**	**Concentration (cells/mL)**	*k* * (div/day)	**Hour 0 viable cells added**	**Hour 20 at bottom (%)**	**Hour 20 in top (%)**
CCMP 452	-18	0%	99%	45,873				
	1	99%	94%	15,903		13,536	**106%**	**60%**
	2	82%	75%	28,643	0.85	14,159	**73%**	**50%**
	3	58%	96%	36,640	0.36	17,941	41%	36%
	4	54%	94%	49,463	0.43	9,622	**105%**	**22%**
	5	41%	92%	54,027	0.13	10,627	60%	38%
UWC 13.03	-18	0%	98%	18,513				
	1	99%	51%	1,957		8,165	28%	28%
	2	95%	85%	1,487	-0.40	7,644	17%	25%
	3	59%	93%	1,780	0.26	8,116	27%	24%
	4	42%	91%	5,950	1.74	10,481	15%	6%
	5	40%	88%	6,217	0.06	11,919	6%	10%

*Specific growth rates for algal cultures were calculated using the equation: *k* = (log_2_ (N_1_/N_0_))/(t_1_-t_0_).

### Cell division (DNA quantification)

DNA content of recently activated *H. akashiwo* cells was monitored in a separate experiment to identify DNA synthesis as a precursor of cell division. Resting cell induction and activation was completed for triplicate sub-cultures of both strains using the same protocols described above. DNA content of *H. akashiwo* cells was measured immediately after resting cells were signaled to activate, and every hour over a 20 hour period. The relative amount of DNA cell^-1^ was determined by sub-sampling 1 mL of cell culture from the experimental flasks and placing a 495 µL aliquot into a 1.5 mL black microcentrifuge tube containing 5 µL of 50 µM Vybrant DyeCycle Green Stain (Invitrogen Molecular Probes, Carlsbad, CA, USA) working solution. The working solution was prepared by diluting 990 µL 5 mM Vybrant DyeCycle Green Stain with 10 µL f/2-Si. A 500 µL aliquot of cell culture was placed into an empty black microcentrifuge tube to serve as an unstained control. The microcentrifuge tubes were inverted 4 times and incubated for 30 min. The stained and unstained cells were transferred into 5 mL glass tubes for flow cytometric measurements. The Vybrant DyeCycle Green stained samples and unstained controls were excited at 488 nm with emission detected at 533 nm (±30 nm) by the Accuri flow cytometer. To determine the average DNA content per cell at each time point, the mean background fluorescent signal (FL1-A) of unstained samples was subtracted from the mean fluorescent signal of stained samples. The mean signal was standardized by dividing each value by 1x10^5^.

### Cell lipid and fatty acid content

Flow cytometric measurements of cell neutral lipid content were made by placing 990 µL aliquots of subsampled cell culture into a 1.5 mL black microcentrifuge tube containing 10 µL 1.25 µM BODIPY 505/515 solution. The solution was prepared by first dissolving 10 mg BODIPY 505/515 stock powder (Invitrogen Molecular Probes, Carlsbad, CA, USA) with 8.06 mL 99% DMSO to make a 5mM BODIPY 505/515 working solution, and then 30 µL of the 5mM working solution was diluted with 10 µL f/2-Si medium. The microcentrifuge tubes were inverted 4 times and incubated for 30 min at 20°C. A 500 µL aliquot of cell culture was placed into an empty black microcentrifuge tube to serve as an unstained control. Stained and unstained cells were transferred into 5 mL glass tubes, and flow cytometric measurements were made in the dark following the same protocol described above. The BODIPY 505/515 stained samples and unstained controls were excited at 488 nm and emission was detected at 533 nm (±30 nm).

Fatty acid profiles for vegetative and resting cells were obtained in a separate experiment using a GC-MS sub-microscale method [39]. Triplicate sub-cultures for CCMP 452 and UWC 13.03 *H. akashiwo* strains were grown to a concentration of ~1x10^5^ cells mL^-1^ and induced into the resting stage as described above. Vegetative cells were sampled immediately prior to resting cell induction. 100 mL samples were collected from each culture flask, placed into screw-threaded clear glass centrifuge tubes (Fisher Scientific, Hampton, NH, USA), and centrifuged at 5000 rpm for 20 minutes at 4°C in a Sorvall RC-5 superspeed refrigerated centrifuge (Fisher Scientific). The cell pellets were resuspended with 250 µL f/2-Si medium, combined into a single 30 mL centrifuge tube and immediately flash frozen in liquid nitrogen and stored at -80°C until lyophilization. The triplicate samples were lyophilized using a Labconco Freezone 2.5 (Labconco, Kansas City, MO, USA) over a 48 hour period. Sample tubes were capped and stored at -20 °C. The tubes were allowed to come to room temperature prior to analysis.

The cell pellets were processed and analyzed following the GC-MS sub-microscale protocol [39]. Briefly, samples were standardized by adding a surrogate mixture with known concentration of C11:0 and C17:0 triglycerides to each sample tube. Each sample was transesterified using methanolic boron trifluoride, followed by a two-step phase separation (brine and isooctane) to extract the fatty acids. The isoocatane layer was transferred to a sample tube and an internal standard of deuterated aromatics was added (“Revised SV Standard”, Restek, Bellefonte, PA, USA). The samples were immediately analyzed using a HP 5890 gas chromatograph equipped with an HP 5971A mass spectrometer (Agilent) and lipid quantification was performed against a 27-component external standard (“NLEA Fame Mix”, Restek, Bellefont, PA, USA). Two separate runs of this experiment were conducted.

### Video capture of cell swimming behaviors

Video observations of cell movements occurred immediately after the resting cells were exposed to the activation conditions (day 1 at L6; filming hour 0) and continued for 5 consecutive days (Figure 2). Video sequences of cell movements were captured to a computer at 10 frames s^-1^ using a Logitech Quickcam Pro900 (infra-red (IR) filter and stock lens removed) that was in a modified housing equipped with a Nikon Nikkor 60 mm lens (aperture f/8) and a #8 extension tube. Cells were observed under dark field illumination using an IR (960 nM) LED bank. The camera and light sources were mounted in a fixed orientation on a computer-controlled motorized platform that moved horizontally and vertically across the tank, to ensure lighting and imaging were consistent at all positions in all replicate filming chambers. The experimental filming tank and automated camera system was located within a temperature- and light-controlled environmental chamber. The environmental chamber was set to the activation conditions described above. A SmartRelay controller (6bit Inc., UT, USA) was used to control camera position, filming duration, and lighting from a computer located outside of the environmental chamber.

Cell movement behaviors were observed at four vertical positions within each replicate chamber. The camera was positioned approximately 100 mm from the tank, providing a field of view (FOV) of approximately 9 mm x 12 mm. The bottom FOV extended upward 2 mm from the base of the tank. The below-halocline FOV was centered about 100 mm above the base. The in-halocline FOV was centered 200 mm above the base. The above-halocline FOV was centered 40 mm above the halocline (240 mm above the tank base). Video clips of 120 seconds duration were captured for each FOV in each replicate chamber at 1 hour intervals over a 19 hour period (hour 0 – hour 19) on each day of observation. The data reported here represent 40 video clips analyzed from the first two days of observation (day 1 and day 2).

### Video processing and swimming path analysis

Pixel positions of the cells in each video clip were determined by video processing using a modified version of the open source video editing package, Avidemux 2.4, to remove background, equalize lighting, threshold frames and set the particle size range to clearly resolve cells [38]. Video was calibrated using a uniform grid of dots with 2.5 mm spacing. Pixel coordinates of cells were converted into physical units and assembled into 2D cell swimming trajectories with Tracker3D, a MATLAB-based motion-analysis package to track organism movement (Grünbaum, unpublished data.). High frequency noise was removed from cell trajectories using a short-interval smoothing spline [23,40]. This spline had knot spacing of 15 frames (1.5 seconds), which was short enough to accurately resolve oscillations in cell trajectories [38]. Overall direction was defined using a long-interval spline with knot spacing of 150 frames (15 seconds) to follow the central axis of each cell’s path. Analysis included only cell trajectories longer than 240 frames (24 seconds).

The splined trajectories were used to characterize cell swimming behaviors. Three swimming metrics (mean vertical velocity, mean horizontal velocity and gross speed) were calculated, as described by Tobin et al. [38]. Gross speed was defined as the total two-dimensional movement along the central axis of a trajectory. Net vertical velocity was defined as the average velocity in the vertical over the length of the trajectory. Mean path statistics were weighted by path duration to avoid biasing in favor of shorter or longer paths.

## Results

### Cell viability and population growth

Strain-specific differences in survivorship and resting cell activation rates were observed in the experimental flasks as *H. Akashiwo* cells transitioned between benthic and pelagic life-stages. After the 18-day induction period, 99% of cells in both strains had entered the resting stage and no motile cells were observed (Table 1). Survivorship during resting cell formation was higher for CCMP 452 than for UWC 13.03. Mean cell concentrations within the experimental sampling flasks decreased by 65% for CCMP 452 and 89% for UWC 13.03, and resting cell viabilities were 94% and 51%, respectively. CCMP 452 cells had a faster rate of activation from the resting stage. When cells were signaled to activate, 17% (CCMP 452) and 4% (UWC 13.03) activated within 24 hours. However, an equal proportion (60%) of both cell populations activated by the end of the study.

The two *H. akashiwo* strains displayed different rates of population growth within the experimental flasks immediately following activation (Table 1). Within 24 hours, CCMP 452 had a positive population growth rate (0.85 div day^-1^) while the UWC 13.03 population continued to decline (-0.40 div day^-1^). However, the UWC 13.03 culture had a sudden increase in the population growth rate (1.74 div day^-1^) after 72 hours under activation conditions (day 4). Although the daily population growth rates differed between the two strains, the average population growth rate over the 5 days under activation conditions was nearly equal for CCMP 452 (0.44 div day^-1^) and UWC 13.03 (0.41 div day^-1^) cells.

Cell counts from the top layer and bottom 10 mL of each water column within the experimental tank provided estimates of vertical cell distributions. The mean proportion of cells in the top layer and bottom 10 mL at end of each observation 20-hour period was generally less than 100% (Table 1). For three days of observation (days 1, 2 and 4) the mean proportions cells in the two layers was greater than 100% for CCMP 452, indicating that more cells were in the chambers at the end of the observation period than had been initially added. This increase suggested that cell division had occurred within the experimental tank.

### Cell division

**Figure 3:**
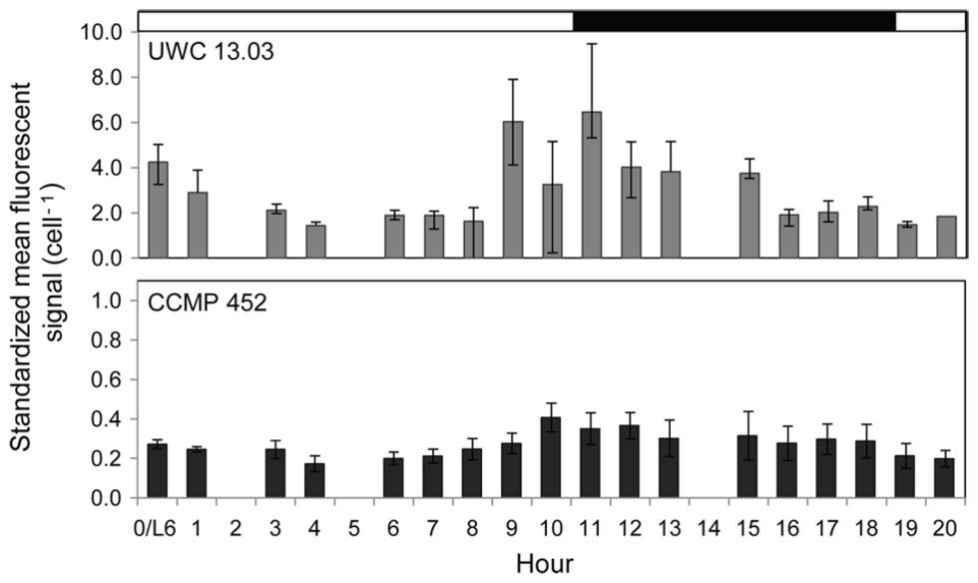
Mean DNA signal cell^-1^ following restoration of “activation” conditions for both *H. akashiwo* strains. Hour 0 indicates the signal expressed by resting cells just prior to exposure to the growth supporting, activation conditions. The triplicate cell culture flasks were unwrapped and exposed to activation conditions at L6 (see text and Figure 2) of the 16 hour light: 8 hour dark photoperiod. Range in sample size (cell #): 3,040 -11,770 (UWC 13.03) and 5,626 -14,918 (CCMP 452). Errors bars show the standard error around the mean.

A Vybrant DyeCycle Green DNA assay provided evidence suggesting that recently activated cells in the experimental flasks may be able to divide within a few hours after exposure activation conditions. Change in the fluorescent DNA signal cell^-1^ appeared to follow a circadian rhythm, increasing and subsequently decreasing in strength for both *H. akashiwo* strains over the first photoperiod under activation conditions. The lowest fluorescent signal was observed 4 hours after resting cell activation was initiated. It then increased at least 2-fold during the transition from the light to the dark period and declined again within the 20 hours of observation (Figure 3). This change in the DNA fluorescent signal cell^-1^ indicated that DNA synthesis was occurring and oscillation in DNA concentration cell^-1^ supports the view that cells were dividing. While the overall pattern of change in the fluorescent signal was similar for both strains, the relative strength of the signal differed between strains (UWC 13.03 was nearly an order of magnitude greater than observed for CCMP 452).

### Fatty acid and neutral lipid content

**Figure 4:**
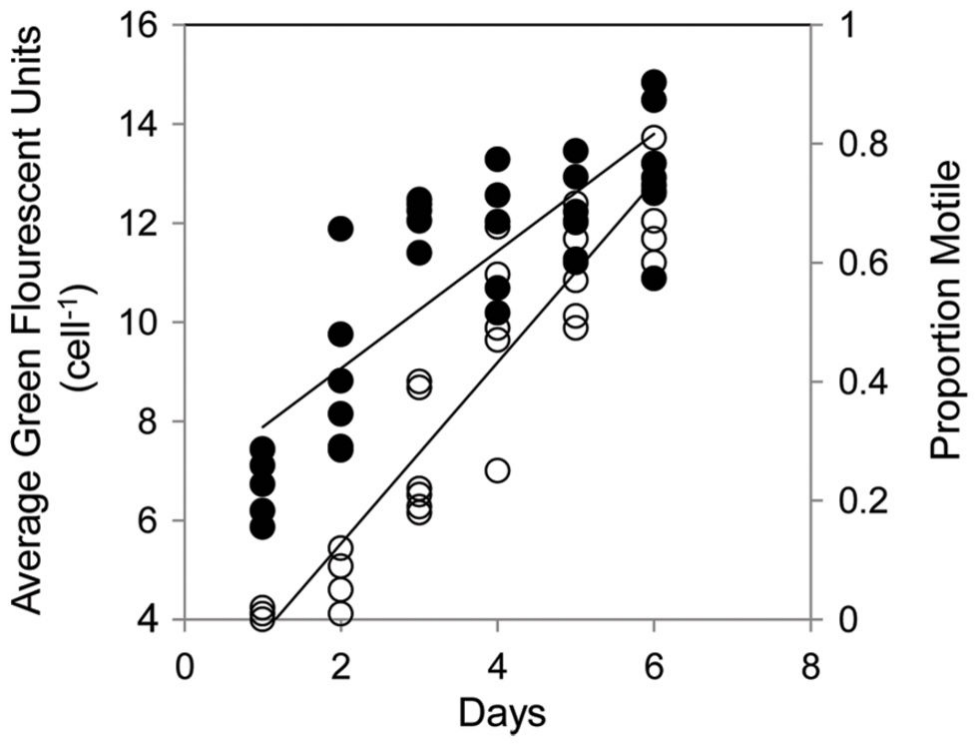
Changes in neutral lipid content cell^-1^ and cell motility following exposure to activation conditions. The scatter plot shows the association of average neutral lipid content cell^-1^ (BODIPY 505/515 signal; black circles) and the proportion of motile cells (open circles) with time under activation conditions (in days) for both *H. akashiwo* strains. A Spearman’s rank correlation indicates that mean neutral lipid content (r = 0.676) and population motility (r = 0.824) both have significant, positive correlations with time under activation conditions (N = 65, p = < 0.001). Daily sample size (cell #): day 1 = 5358, day 2 = 9,039, day 3 = 11,526, day 4 = 16,624, day 5 = 18,073, and day 6 = 9,480.

To examine lipid reserves in vegetative and resting life-stages, neutral lipid and total fatty acid content in *H. akashiwo* cells within the experimental culture flasks were measured using the fluorescent dye BODIPY 505/515 and GC-MS, respectively. Mean neutral lipid content cell^-1^ showed a greater than 2-fold decline as cells entered the resting stage. Both neutral lipid content and cell motility were tightly associated with cell state for both strains of *H. akashiwo* (Figure 4). A Spearman’s rank correlation showed that both mean neutral lipid content cell^-1^ and population motility had a significant, positive correlation with time under activation conditions (r = 0.676 and 0.824, respectively, N = 65, p = < 0.001), indicating that cell swimming capacity and lipid synthesis are quickly and synchronously restored upon activation from the resting stage. GC−MS analysis showed a significant change in total fatty acid cell^-1^ as both CCMP 452 and UWC 13.03 cells entered into the resting stage. Resting cells had significantly less total fatty acid cell^-1^ than vegetative cells (Student’s t-test: N = 3, p < 0.05). This result was observed for both strains in the two independent experimental runs. Vegetative cells of UWC 13.03 had 137.1±30 pg cell^-1^ (run 1) and 376.1±106 pg cell^-1^ (run 2), and resting cells had 18.2±3 pg cell^-1^ (run 1) and 23.4±4 pg cell^-1^ (run 2). For CCMP 452, vegetative cells had 68.5±15 pg cell^-1^ and 108.3±19 pg cell^-1^, and resting cells had 35.4±12 pg cell^-1^ and 22.3±3 pg cell^-1^ for runs 1 and 2, respectively. These values represent a decrease of 87% (run 1) and 94% (run 2) for UWC 13.03, and 48% (run 1) and 79% (run 2) for CCMP 452. Although, GC-MS analysis shows that UWC 13.03 vegetative cells have higher total fatty acid content than vegetative CCMP 452 cells, the UWC 13.03 cells lost significantly more fatty acid content during resting cell formation. As a result, the resting cells of both strains had similar amounts of total fatty acid.

**Figure 5:**
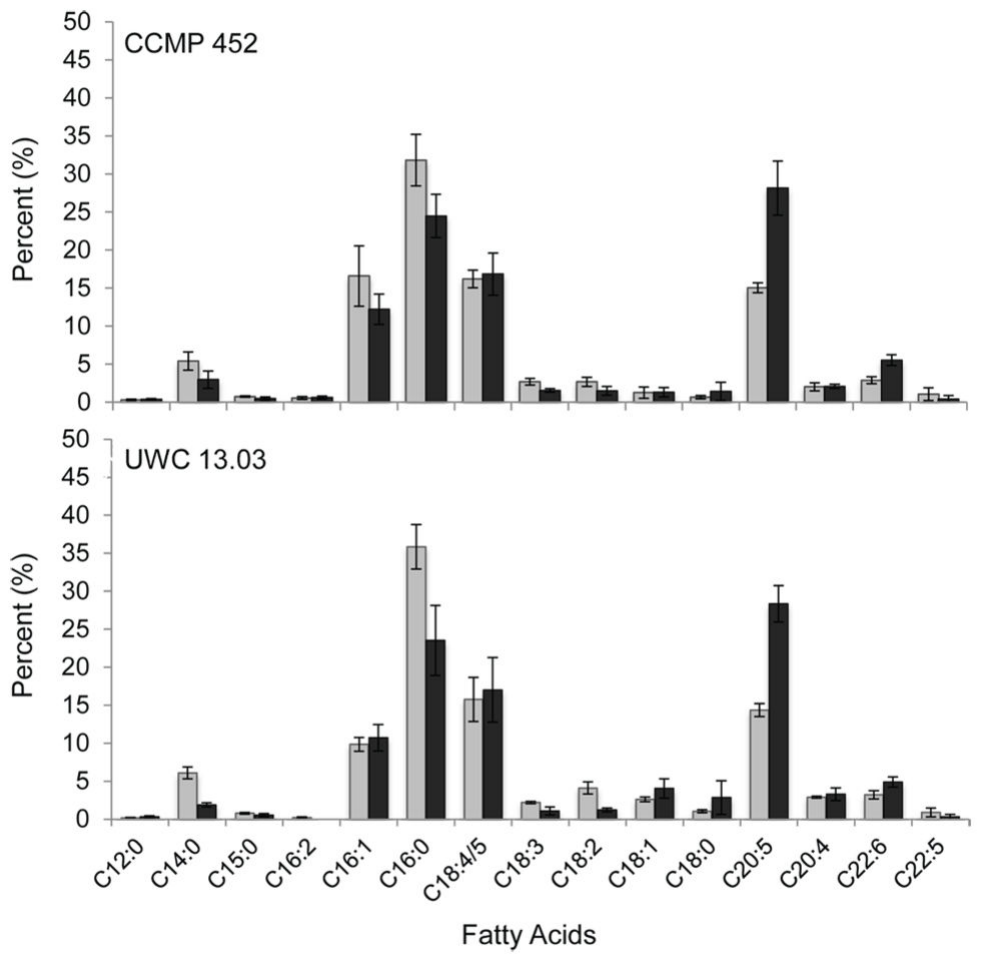
Fatty acid profiles of vegetative and resting *H. akashiwo* cells. The bar graphs show the average proportion (N = 3) of each fatty acid based on the total fatty acid content for strains CCMP 452 (top) and UWC 13.03 (bottom), during vegetative (grey bars) and resting (black bars) life-stages. Error bars represent the standard deviation around the mean.

To assess whether fatty acid composition shifts between vegetative and resting life-stages, total fatty acid profiles of CCMP 452 and UWC 13.03 were compared. A broad distribution of fatty acids from C12:0 to C 22:6 was observed (Figure 5). In the vegetative stage, predominant fatty acids included C14:0, C16:0, C16:1, C18:4/5 and C20:5. Shifts in fatty acid profiles of resting cells were similar for both *H. akashiwo* strains. Resting cells showed either a decline or no change in the relative proportion of most fatty acids types, including predominant vegetative fatty acids C16:0, C16:1 and C18:4/5. However, a significant increase in PUFAs C20:5 and C22:6 was observed, indicating that the relative proportion of long-chain unsaturated fatty acids increased in resting cells compared to vegetative cells (Figure 5).

### Cell swimming behaviors and vertical distributions

**Figure 6:**
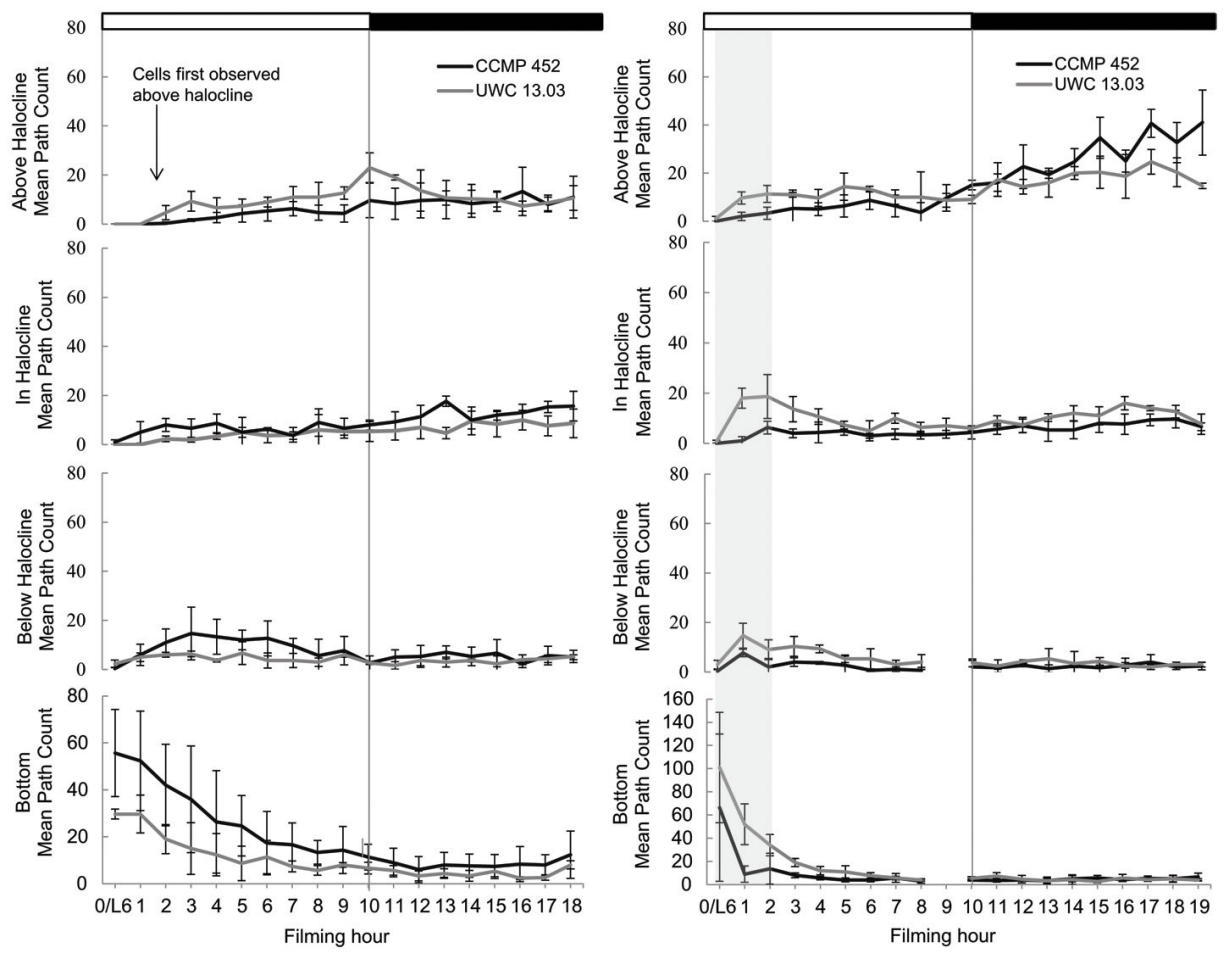
*H. akashiwo* cell distributions within the experimental tank. Distributions are based on the mean path count in each field of view for day 1 (left) and day 2 (right) filming observations. The grey shading on the day 2 plot represents vegetative cells determined to have previously activated on day 1. Error bars show the standard deviation around the mean.

Video observations revealed that emergence from the resting stage occurred rapidly after resting cells were signaled to activate. Within one hour, swimming cells of both strains were observed in the in below-halocline and in-halocline FOVs. However, UWC 13.03 cells were observed above the halocline 1-2 hours earlier than CCMP 452 cells, indicating that UWC 13.03 cells were able to cross the halocline before CCMP 452 cells (Figure 6, day 1). Decreasing (but non-zero) swimming path counts within the bottom FOV over time indicated that newly emerged cells continued to leave the bottom of the chamber and swam up into the water column throughout the light period (Figure 6).

Cell distributions within the experimental tank were similar on both days of video observation. However, the flux of cells into the water column appeared to be higher on day 2 than on day 1 (Figure 6, day 2 - shaded region). Microscope-based observations revealed that all resting cells within the experimental culture flasks did not activate at the same time, indicating previously-emerged vegetative cells co-occurred with resting cells in the experimental culture flasks on days 2-5. Hence, cells added to the tank on day 2 included both life-stages, and the greater flux of cells observed within the first two hours on day 2 could be accounted for by motile cells that had emerged on the previous day.

To distinguish between these co-occurring cell types in our video observations, we classified cell swimming trajectories as “newly emerged” or “previously emerged” based on the time period over which the swimming paths were observed. All swimming paths observed on day 1 were classified as “newly emerged”. On day 2, swimming paths observed in the water column during the first two hours of observation were classified as “previously emerged”, and paths observed during the rest of the filming period (hours 3-20) were classified as “newly emerged”.

**Figure 7:**
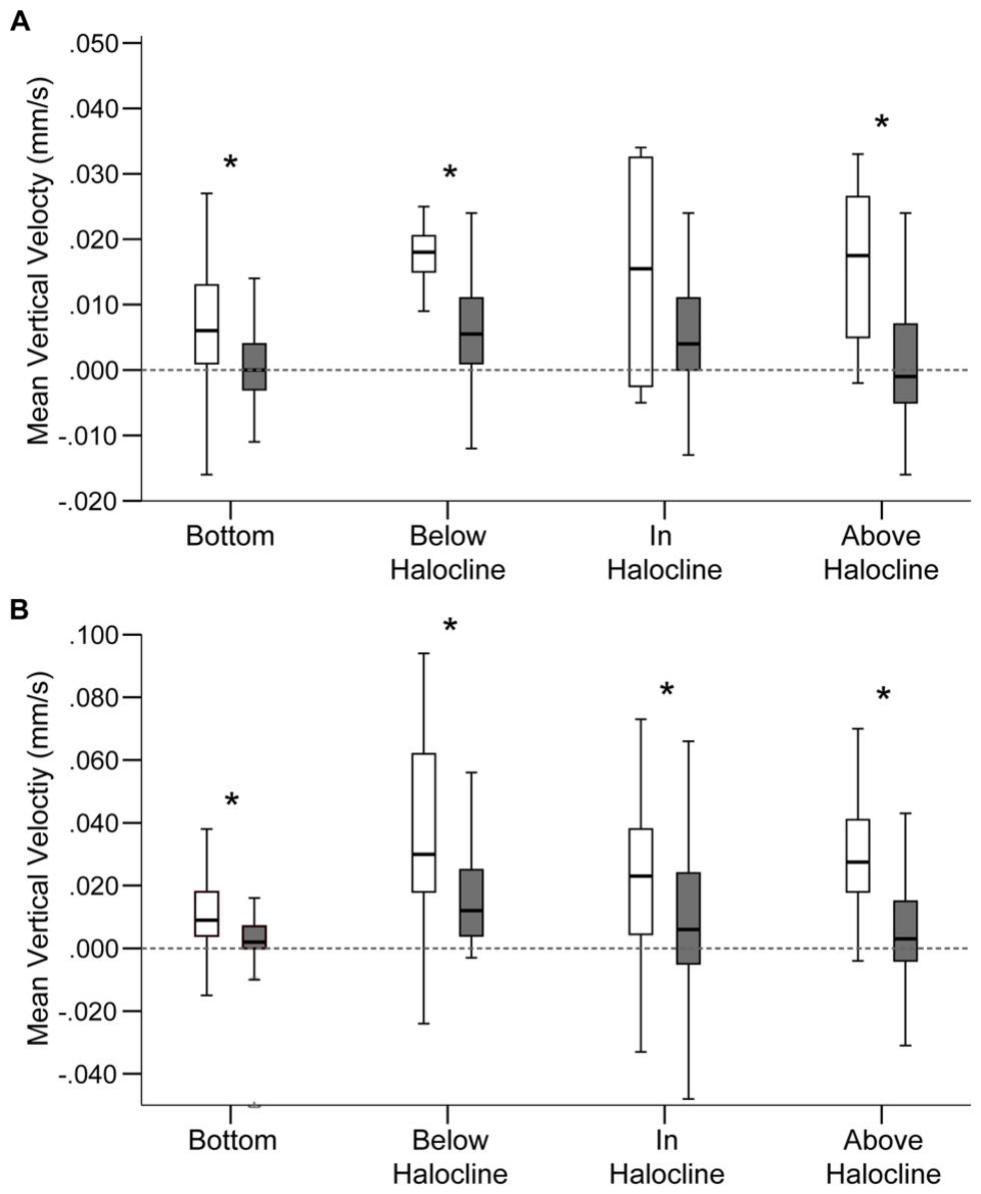
Vertical velocities observed for emerged *H. akashiwo* cells. Boxplots show the range of vertical velocities for “newly emerged” cells (■) and “previously emerged” (□) cells in each field of view for (**A**) CCMP 452 and (**B**) UWC 13.03. Asterisks indicate significantly different (p-value < 0.05) vertical velocity distributions based on a Two-sample Kolmogorvo-Smirnov test (see Table 2 for test statistics). Note the 2-fold difference in the *y*-axis scale between the two algal strains.

Vertical velocity distributions were significantly different between the two cell classifications in all FOVs except the in-halocline FOV for CCMP 452 (Two-sample Kolmogorvo-Smirnov test, Table 2; Figure 7). For both strains, previously emerged cells had faster net upward swimming velocities (CCMP 452 = 19.3 µm/s, UWC 13.03 = 35.3 µm/s) compared to newly emerged cells (CCMP 452 = 7.4 µm/s, UWC 13.03 = 11.4 µm/s).

**Table 2:** Test statistics based on a Two-sample Kolmogorvo-Smirnov test (α = 0.05) for differences in median vertical velocities between “newly emerged” and “previously emerged” cells.

**Strain**	FOV*	**Z**	**P-value**	**N Previously Emerged**	**N Newly Emerged**
CCMP 452	Bottom	5.11	< .001	226	1403
	Below Halocline	3.57	< .001	24	526
	In Halocline	0.98	0.292	4	603
	Above Halocline	1.7	0.006	8	1312
UWC 13.03	Bottom	7.15	< .001	459	958
	Below Halocline	3.89	< .001	53	471
	In Halocline	3.53	< .001	56	1071
	Above Halocline	4.09	< .001	32	1346

* FOV = field of view.

**Figure 8:**
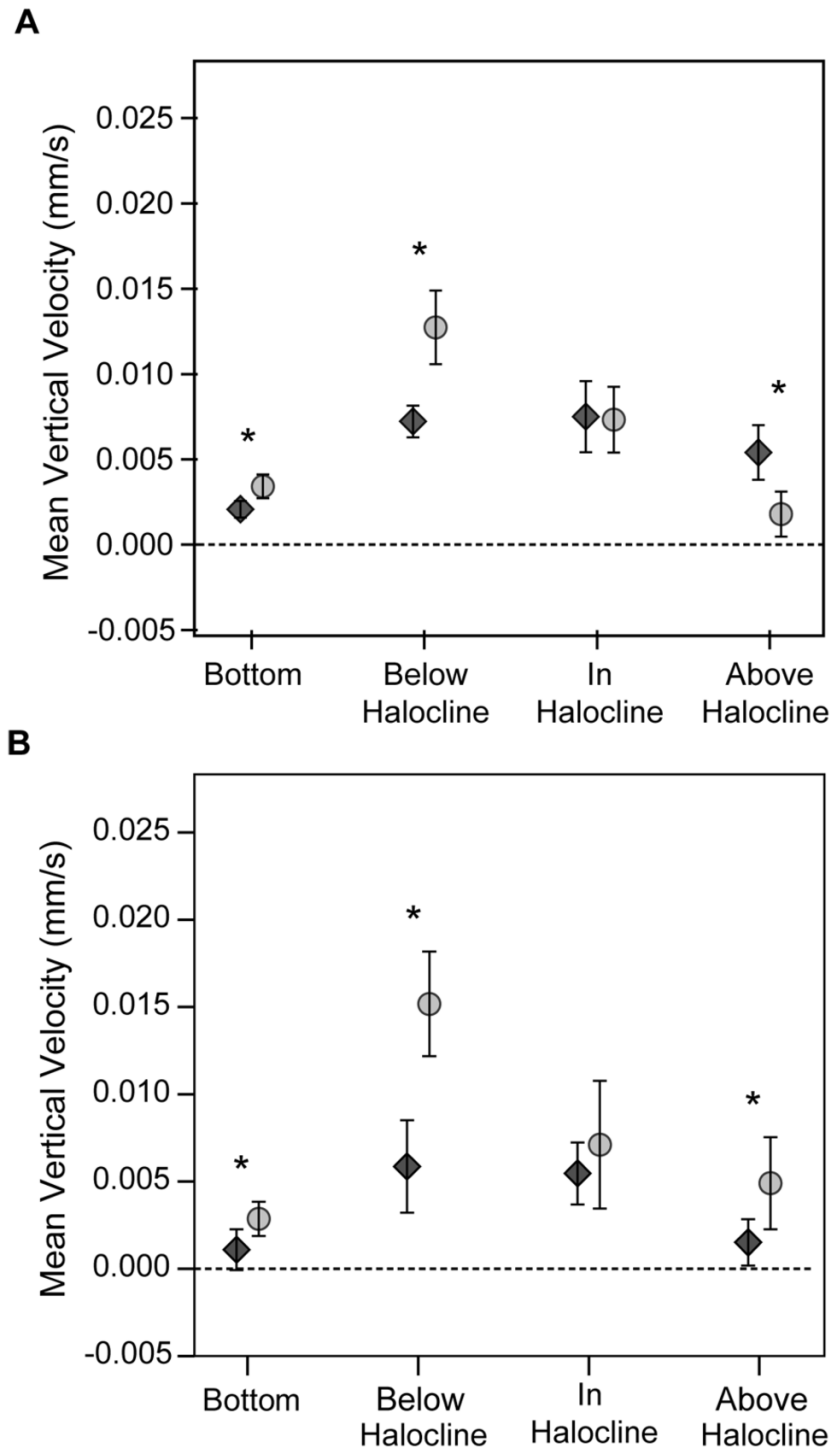
Mean vertical velocities of newly emerged *H. akashiwo* cells. The mean vertical velocity is plotted at each field of view for day 1 (A) and day 2 (B) for UWC 13.03 (grey circles) and CCMP 452 (black diamonds). Error bars show 95% confidence intervals. Asterisks indicate statistically significant differences in median vertical velocity based on a Mann Whitney U test – (**A**) BT: Z = -5.00, p < 0.001, N = 565, 1130 (UWC 13.03, CCMP 452), BH: Z = -4.12, p < 0.001, N = 231, 414 (UWC 13.03, CCMP 452), IH: Z = -1.65, p = .100, N = 517, 296 (UWC 13.03, CCMP 452), AH: Z = -4.31, p < 0.001, N = 352, 1130 (UWC 13.03, CCMP 452), (**B**) BT: Z = -3.70, p < 0.001, N = 879, 499 (UWC 13.03, CCMP 452), BH: Z = -3.67, p < 0.001, N = 293, 136 (UWC 13.03, CCMP 452), IH: Z = -1.26, p = .206, N = 610, 311 (UWC 13.03, CCMP 452), AH: Z = -2.93, p = .003, N = 823, 968 (UWC 13.03, CCMP 452).

Newly emerged UWC 13.03 cells generally exhibited faster swimming rates than newly emerged CCMP 452 cells. The maximum gross speed observed for UWC 13.03 was 90 µm s^-1^ and the maximum upward velocity was 87 µm s^-1^, more than two times greater than for CCMP 452 (45 µm s^-1^ and 36 µm s^-1^, respectively). Comparisons of mean vertical velocities (Figure 8) showed UWC 13.03 had significantly faster upward vertical velocities, 12.7 µm s^-1^ on day 1 and 15.2 µm s^-1^ on day 2, than CCMP 452 (7.2 µm s^-1^ and 5.9 µm s^-1^, respectively) prior to reaching the halocline (Mann-Whitney U test). However, mean vertical velocities were not statistically different between the two strains at the halocline, indicating that the presence of the halocline reduced vertical velocities of UWC 13.03 cells.

**Figure 9:**
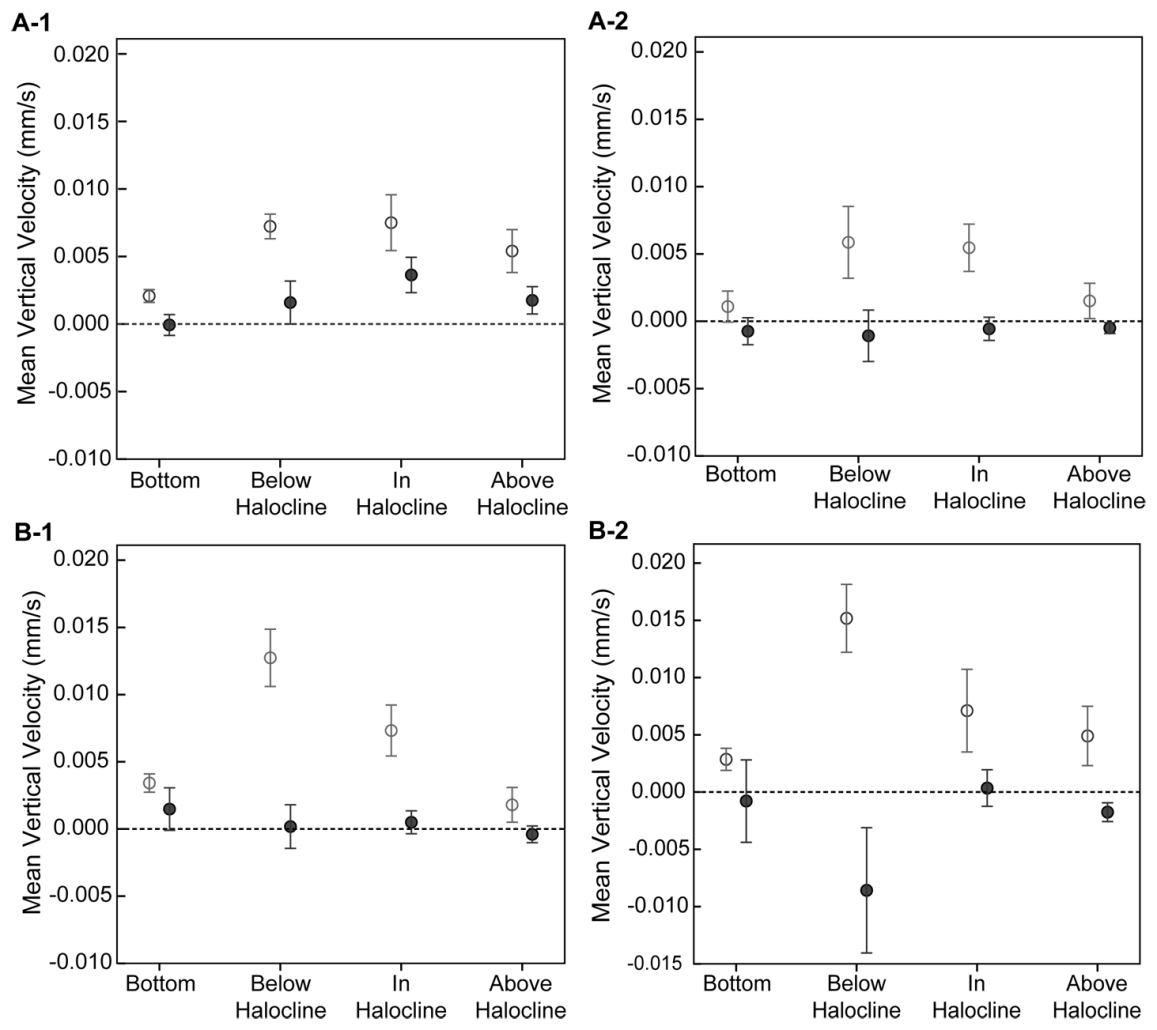
Light and dark comparisons of *H. akashiwo* mean vertical velocities. Mean vertical velocities are plotted for light (open circle) and dark (black circle) photoperiods for CCMP 452 (**A**) and UWC 13.03 (**B**) on day 1 (1) and day 2 (2) for each field of view. Error bars show the standard error around the mean. See Table 3 statistics for the Chi-square Test for comparison of proportions (Χ^2^).

Newly emerged *H. akashiwo* cells expressed different swimming behaviors in light vs. dark periods. In both strains, net upward vertical velocities were observed in the light period, but in the dark period net vertical velocities were generally near zero (Figure 9). The proportion of upward- and downward-directed swimming cells differed between the light and dark periods (Chi-square test for comparison of proportions, test statistics: Table 3). In both CCMP 452 and UWC 13.03, 60-70% of swimming cells in the water column had upward-directed swimming during the light period, while the proportions of upward and downward swimmers were nearly equal during the dark period (Figure 9).

**Table 3:** Proportions of upward and downward directed swimming cells during the light and dark periods for the two days of observation.

			**Day 1**				**Day 2**				
**Strain**	**Photo Period**	**N**	**Up (%)**	**Down (%)**	**Χ^2^**	P-value*	**N**	**Up (%)**	**Down (%)**	**Χ^2^**	P-value*
CCMP 452	Light	1446	69	31			497	60	40		
	Dark	746	56	44	36.5	< 0.001	1155	44	56	32.2	< 0.001
UWC 13.03	Light	1095	69	31			992	73	27		
	Dark	773	44	56	118.0	< 0.001	1013	46	54	147.8	< 0.001

* Significant differences in swimming direction between the light and dark were determined using a Chi-square Test for comparison of proportions (Χ^2^).

## Discussion

Quantifying physiological and behavioral characteristics that influence algal cells’ rates of benthic emergence and vertical fluxes to the upper water column is critical for understanding the basic timescales over which *H. akashiwo* harmful algal blooms form. A key finding was that cells of *H. akashiwo* can rapidly emerge from the benthic resting stage, regain the swimming capacity necessary to cross environmentally relevant haloclines, and undergo cell division within hours after growth supporting conditions are restored. We also observed pronounced strain-specific variation in important physiological and behavioral traits, including survivorship during life-stage transitions and post-transition specific growth rates and swimming behaviors. Collectively, these traits function as strain-specific “population growth strategies” that govern timescales of potential *H. akashiwo* bloom formation.

### Strain-specific variation in life-stage transitions and growth

Greater cell survivorship and activation rates suggest that CCMP 452 was more successful at transitioning into and out of the benthic resting stage than UWC 13.03. UWC 13.03 had significantly higher mortality during life stage transitions and exhibited a slower rate of activation compared to CCMP 452. These results suggest that strains of *H. akashiwo* which are more successful at transitioning between life stages (e.g., CCMP 452) could supply a higher inoculum of vegetative cells into the water column, compared to strains with reduced survivorship (e.g., UWC 13.03).

Population growth rates immediately after activation from the resting stage also differed between the two strains. The CCMP 452 population density nearly doubled within 24 hours of exposure to activation conditions, while UWC 13.03 showed an initial decline. Although daily population growth rates differed between the two strains, their average population growth rates over the 5 days of observation were nearly equal. This outcome suggests that initial growth responses may not be representative of long-term population growth. Our average population growth rate (0.40 div d^-1^) is consistent with long-term growth rates observed for vegetative cells of both *H. akashiwo* strains under the environmental conditions tested in this study (E.T., unpublished data).

Two lines of evidence suggest that *H. akashiwo* cells were able to undergo cell division rapidly − within 24 hours − after activation from the resting stage. First, flow cytometry cell counts from the subsamples collected from both the experimental flasks and the video observation tank showed that more CCMP 452 cells were present after 24 hours then were originally present (see Table 1, day 1). Second, DNA analysis indicated that DNA synthesis − a precursor to cell division – may have occurred in both strains of *H. akashiwo*. The approximately 2-fold increase in the DNA signal per cell observed near the light/dark transition for both strains provides preliminary evidence that a subset of the cell population was preparing to divide. Cell size is often used in combination with DNA content to infer that cell division has taken place. Unfortunately, since the sampled *H. akashiwo* cultures contained mixed life stages (both resting and vegetative), the cell size data in our observations were not statistically informative.

We observed that DNA dye signal strength differed between strains. The DNA signal cell^-1^ was nearly an order of magnitude greater in UWC 13.03. The reason for this variance in the relative signal strength is not presently known. Preliminary experiments suggest that distinct strains of *H. akashiwo* may have dissimilar levels of mitochondrial DNA (Black and Cattolico, unpublished data). Whether the total DNA complement also varies among strains is currently under investigation. Alternatively, unidentified strain-specificity in fluorochrome uptake or variation in the number of dead or dying cells within the cell cultures could have affected the relative fluorescent intensities [41].

The cell cycle in vegetative H. *Akashiwo* displays a circadian rhythm that is regulated by photoperiod [32,42]. Satoh et al. reported that *H. akashiwo* (strain NIES 6) required a minimum 6 hour light period for cell division to occur [42]. If this requirement applies across strains and physiological states, then our experimental post-emergence conditions were suitable for rapid onset of division. It is unclear why population growth lagged in UWC 13.03 despite the evidence for DNA synthesis. A possible explanation is that recently activated cells advanced through S phase but failed to complete cell division. This interpretation of weakened physiological condition associated with life-stage transitions is consistent with our observations of higher mortality in UWC 13.03.

### Role of neutral lipids in life-stage transitions and motility

Neutral lipid and fatty acid content per cell in *H. akashiwo* changed with physiological state. Both BODIPY 505/515 neutral lipid and GC-MS assays demonstrated that *H. akashiwo* resting cells have significantly less total lipid content than vegetative cells. UWC 13.03 cells lost significantly more fatty acid content during resting cell formation than CCMP 452. Consequently, cells of both strains had a similar amount of total fatty acid (~20-30 pg cell^-1^) when they reached the resting state. It is possible this fatty acid content approximates a minimum threshold required to ensure survival during and emergence from the resting stage.

We observed shifts in fatty acid composition between the two life-stages. In both strains, the relative proportion of long-chain fatty acids was higher in resting cells compared to vegetative cells, suggesting that *H. akashiwo* cells appeared to selectively retain some PUFAs over other fatty acid types during resting cell formation. A significant change was particularly noted for eicosapentaenoic acid (C20:5), a fatty acid found in unusually high amounts in *H. akashiwo* cells [43]. This long chain fatty acid represented approximately 15% of the total fatty acids in vegetative cells, increased to approximately 28% of the total fatty acids in resting cells. Our results are consistent with the hypothesis that fatty acid composition plays an integral role in successful algal life stage transitions. PUFAs are particularly important for building photosynthetic membranes [44] and are necessary for modification of cellular and organelle membranes during changes in environmental conditions [14,45,46]. For example, Eicosapentaenoic acid has been hypothesized to contribute to the maintenance of “optimal membrane fluidity… under low temperature or high hydropressure conditions” [47]. Retention of PUFAs in *H. akashiwo* resting cells may represent a more generalized response to decreased temperature that is often associated with the benthic environment, thus further promoting survival while the alga is in a metabolically down-regulated state (Deodato and Cattolico, unpublished data).

It is generally recognized that neutral lipids serve as energy stores available for maintaining critical metabolic processes [14,48], suggesting they may be important energy sources for cell motility. We found that both neutral lipid content and motility have a positive association with activation from the resting stage. Consistent with our findings, close associations between lipid content and cell motility have been reported in zoospores of the kelp, *Pterygophora californica* [49] and in multiple strains of the dinoflagellate, *Karenia brevis* [50]. Although, we cannot determine whether lipids are being catabolized to provide energy for swimming, our data do indicate that endogenous lipid stores and motility are closely coupled in time.

### Swimming behaviors during benthic-pelagic transition

Increased temperature (12°C) and light (20 µmol m^-2^ s^-1^) triggered *H. akashiwo* resting cells to transition out of the benthic stage and to regain motility. Our video observations revealed that cells of both strains began to swim up into the water column within an hour after these growth supporting conditions were restored. Newly emerged UWC 13.03 cells exhibited significantly faster mean upward swimming velocities prior to reaching the halocline than newly emerged CCMP 452 cells. This observation is consistent with previous studies on *H. akashiwo* swimming behaviors. Bearon et al. (2004) reported differences in gross swimming speeds for geographically distinct strains of *H. akashiwo*: CCMP 452 (49–66 µm s-1) and CCAP 934-1 (88–119 µm s-1). Helical swimming modes were different across five strains of *H. akashiwo* based on 2D observations and 3D model simulations [51]. Such interstrain variations in *H. akashiwo* swimming behaviors likely correspond to morphological and/or physiological differences, but these have not yet been identified.

Newly emerged *H. akashiwo* cells quickly reacquired swimming capacity sufficient to cross an environmentally relevant halocline (a 13 psu salinity jump). UWC 13.03 cells showed a significant decrease in mean vertical velocity at the halocline, so that both strains had similar mean vertical velocities within the halocline (see Figure 8). However, UWC 13.03 cells were nonetheless observed above the halocline 1-2 hours before CCMP 452 cells. This result indicates that more vigorous upward swimming by UWC 13.03 cells prior to reaching the halocline enabled them to transit the entire experimental water column more quickly than CCMP 452 cells. Differences in time needed for cells of each *H. akashiwo* strain to reach near-surface layers could be greatly magnified when emergence occurs at the greater depths typical of natural habitats.

Both *H. akashiwo* strains changed mean vertical velocity between the light and dark. In the light, cells expressed faster, net upward velocities while net vertical velocities were near zero in the dark. These differences were a result of proportional shifts in swimming direction. More cells swam upward in the light, while the number of upward and downward swimming cells was nearly equal in the dark. Our findings are similar to that of Wada et al. (1985) who reported that *H. akashiwo* (strain OHE-1) had more upward directed movement in the light than in the dark [52]. Contrary to our findings, however, Wada et al. reported a distinct downward vertical migration during the dark period, inferred from cell distributions within unstratified 15 mL tubes. Our video observations, in stratified water columns that suppress water motion, did not indicate a shift in vertical population distribution between light and dark periods.

### Population growth strategies and timescales of *H*. *Akashiwo* HAB formation

Our results show that *H. akashiwo* exhibits pronounced strain-specific variation in key physiological and behavioral traits, including survivorship during life-stage transitions, specific growth rates, and swimming behaviors. Collectively, these traits may function as “population growth strategies” that potentially regulate timescales of *H. akashiwo* HAB formation. The CCMP 452 strain exhibited enhanced survivorship coupled with higher rates of resting cell activation and cell division. This “survive and divide” strategy may indicate that energy reserves are used to enhance cell survival during the benthic resting stage and rapid onset of cell division. The UWC 13.03 strain exhibited reduced survivorship and a lag in growth, but had significantly faster upward swimming behavior following benthic emergence. This “swim-first” strategy may indicate that energy reserves are used to fuel swimming, allowing newly emerged cells to quickly reach near-surface waters.

**Figure 10:**
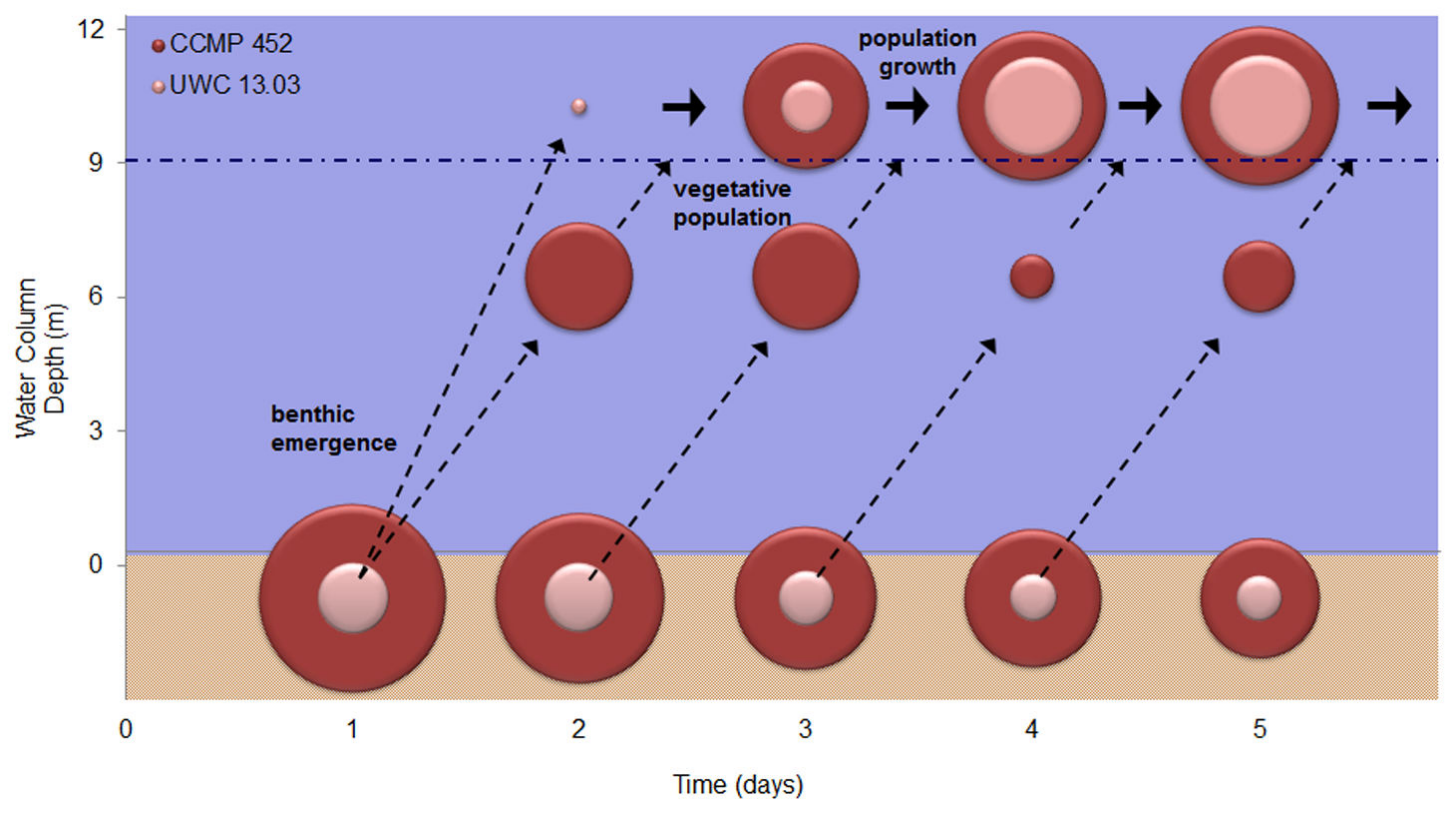
Heuristic model of *H. akashiwo* bloom formation (strains CCMP 452 and UWC 13.03). Observed values of cell viability and rates of resting cell activation, up-swimming and cell division (see Table 1 and Figure 8) were used to calculate benthic and vegetative population densities (bubble plots) and their vertical distributions in the water column (*x*-axis) over time (*y*-axis). In the bubble plot, areas are proportional to population densities. The dashed arrows indicate upward swimming of daily cell “cohorts” leading to aggregation above the halocline (represented by the dot-dashed line at 9 m). Block arrows represent population growth of the near-surface cell aggregates.

To gain insight into how these population growth strategises could regulate timescales of bloom formation, we generated a simple, heuristic model of *H. akashiwo* benthic emergence and near-surface bloom formation (Figure 10). Strain-specific measurements were used to simulate the “survive and divide” (CCMP 452) and “swim-first” (UWC 13.03) population growth strategies, to explore how these strategies influence the rates of near-surface cell accumulations. Prior vegetative populations of CCMP 452 and UWC 13.03 cells were at assumed densities of 5x10^5^ cells L^-1^, a typical *H. akashiwo* HAB density in the Salish Sea. Mortality rates observed during resting cell formation were applied to calculate benthic population densities of viable resting cells. Rates of resting cell activation were then applied over a 4 day period and each “cohort” of cells was tracked as they emerged from the sediments. The transit time (based on the observed mean vertical velocities; see Figure 8) was calculated as cell cohorts swam upward into a 12 m water column, and each cohort’s growth occurred according to the rates (k) measured in this study (see Table 1). Additionally, we assumed that: 1) activated cells immediately and synchronously swim to the surface; 2) the depth of the halocline is fixed; and, 3) cells are retained within the surface layer once they cross the halocline. Mortality rates due to predation or pathogens were neglected. Our simplified model is therefore an examination of population growth potential that may not be realized under real-world conditions.

Under these assumptions, model results suggest that the size of the cell inoculum into the water column − a function of benthic viable resting cell density and activation rate – most strongly determines the timescales over which blooms can potentially form. The “survive and divide” strategy of CCMP 452 facilitated a greater flux of cells into the water column, and subsequently this strain increased in near-surface population density more rapidly than UWC 13.03. Based on our calculations for a 12 m water column, the “swim-first” strategy enabled the UWC 13.03 cells to reach the surface layer in 22 hours, approximately half the time it took CCMP 452 cells (39 hours). However, near-surface accumulation of UWC 13.03 cells occurred at a much slower rate than CCMP 452. He et al. (2008) used a biophysical numerical modeling approach to reach a similar conclusion about the central role of cell inoculum size in bloom dynamics of the harmful dinoflagellate, *Alexandrium fundyense*, in the Gulf of Maine [9].

While our findings clearly demonstrate that physiological and behavioral traits can regulate bloom formation, environmental characteristics that were omitted from our simplified model can also affect *H. akashiwo* HABs timescales. Algal swimming interacts with physical flows to influence cell concentrations and distributions in the water column [25,40,53-57]. For example, cells can be transported by horizontal advection from ambient currents that may vary in speed and direction with depth. Vertical mixing of cells by turbulence also varies with depth, due to boundary-layer effects and interactions with obstacles such as sediments, biota and seabed topography [58-60]. Hence, horizontal and vertical transport processes also strongly affect the time required for cells to migrate through the water column [38]. Biological controls such as predation, competition and viral infection can further limit *H. akashiwo* cell abundance and distribution [61-64].

Our findings suggest that distinct *H. akashiwo* strains utilize diverse population growth strategies that potentially influence bloom dynamics. Such phenotypic diversity is widespread among many species of algae and likely facilitates survival under a wide range of environmental conditions. It is generally understood that algal strains under long-term culture may express traits that are a result of selective pressures experienced under culture conditions [65]. Hence, our observations may not reflect the precise pre-culture characteristics of the observed strains, and do not capture the full diversity of *H. akashiwo* in natural ecosystems. We believe that our data nonetheless provides interesting ecological insights into how *H. akashiwo* strains may express alternate responses to the same environmental signals. Our observations suggest that distinct strategies utilized by *H. akashiwo* strains may reflect habitat-specific trade-offs between physiological and/or behavioral traits. The diversity and potential adaptive value of population growth strategies across geographically distinct strains of *H. akashiwo* are currently unknown but are an important priority for future research.
